# An unusual intracranial metallic foreign bodies and panhypopituitarism

**DOI:** 10.11604/pamj.2014.19.33.3862

**Published:** 2014-09-15

**Authors:** Mohammed Lakouichmi, Hicham Baïzri, Abdelilah Mouhsine, Abderrahmane Boukhira, Ali Akhaddar

**Affiliations:** 1 Maxillofacial Surgery Department, Avicenne Military Hospital, School of Medicine, Cadi Ayyad University, Marrakech, Morocco; 2 Endocrinology Diabetes and Metabolic Diseases Department, Avicenne Military Hospital Marrakech, Marrakech, Morocco; 3 Medical Imaging Department, Avicenne Military Hospital Marrakech, Marrakech, Morocco; 4 Biochemistry Department, Avicenne Military Hospital Marrakech, Marrakech, Morocco; 5 Neurosurgery Department, Avicenne Military Hospital Marrakech, Marrakech, Morocco

**Keywords:** Panhypopituitarism, migraine headaches, intracranial

## Abstract

A 49 years old man, with a history of aggression at the age of 18 years by a pair of scissors, who consulted for unilateral migraine headaches look straight. Paraclinical explorations concluded that trauma to anterior pituitary by a metallic foreign body from the right nostril to the sella, responsible for panhypopituitarism and sinusitis. The headaches are frequent causes of consultation, often treated symptomatically but rarely explored. The direct trauma to the pituitary gland, by a metallic foreign body, is exceptional. We report the case of neglected panhypopituitarism, discovered 31 years after injury with a pair of scissors.

## Introduction

Hemi-cranial headaches are a frequent reason for consultation in medical practice. They are often underestimated and treated symptomatically without any etiological. Direct trauma to the sella, with a metal object, is exceptional. We report the case of a post-traumatic panhypopituitarism discovered 31 years later on the occasion of paroxysmal headache.

## Patient and observation

**Figure 1:**
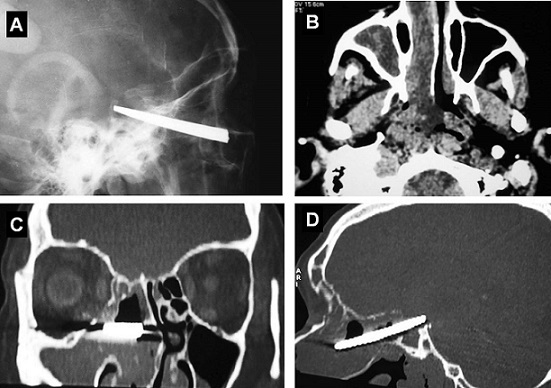
Metallic foreign body from the nasal cavity to the sella ((A), X-ray profile of the skull). Scano-graphic aspects: Filling reaction speed of the maxillary sinus and the ipsilateral nasal cavity with an inflammatory granuloma choanal area and retro-choanal right in axial section ((B) parenchymal window), coronal reconstructions (C) and sagittal (D) (bone windows)

A 49 year-old man, consulted for straight migraine headaches with intermittent ipsilateral rhinorrhea lasting for several years. The general examination found a pale patient, glabrous, very slow, with a sallow complexion of the skin, dry skin, hair removal diffuse, a hoarse voice and hearing loss. The maxillofacial examination objectified a right maxillary sinus syndrome, dominated by unilateral craniofacial pains. In intraoral, he had macroglossia. Radiography of the face, impact Blondeau, showed opacity of the right maxillary sinus with a metallic foreign body. The skull x-ray profile and craniofacial computed tomography (CT-scan) in axial and coronal reconstructions, confirmed the ethmoidal, maxillary and sphenoid sinus reaction, ipsilateral and the presence of a foreign body. It's a metallic foreign body from the right nostril to the sella with a large inflammatory granuloma look (Figure 1). The hormonal test has found a pan hypopituitarism affecting all endocrine axes. The subsequent history of the family members reported the history of aggression by a scissors at the age of 18. The evolution was marked by a progressive deterioration of the general condition of the patient, for 31 years, with no exploration. Given the age of the trauma, the patient refusing any surgery and the surgical risks of a possible extraction of the metal object, abstention was required.

## Discussion

Headaches, banal reason for consultation, cover all medical disciplines. This symptom, very common, should never be underestimated. A clinical examination supplemented by a minimum balance, biological and radiological, is required whenever these pains do not respond to usual treatment. In this observation, the clinical signs of a complete anterior pituitary insufficiency were evident. The metal object caused a direct trauma to the anterior pituitary origin of hypopituitarism. The pituitary post was spared this trauma because the patient did not have diabetes insipidus. The correlation between serious head injury and panhypopituitarism is discussed in the literature, some authors report up to 60% of pituitary deficits subsequent to brain injury [1]. In a review of the literature we found no similar case. However, the association of headache and intracranial metallic foreign body was reported in a man of 22 years [2]. Most often, these head injuries are by trans-orbital and are fatal [3, 4]. And in rare cases, they are made through the fontanels in childhood [2]. In our case the metal object was introduced by the right nasal cavity causing a reaction sinusitis and especially a panhypopituitarism unsubstituted for 31 years. The reaction was responsible for sinusal headaches that were treated symptomatically since the age of 18 without any exploration.

## Conclusion

Mortality in penetrating brain injury is usually high. Survival with panhypopituitarisme caused by a metal object unknown for 31 years is an exception.
